# The Clinical Research of the Chronic Cough After COVID-19 Infection

**DOI:** 10.3390/jcm15062174

**Published:** 2026-03-12

**Authors:** Juan Wang, Lingling Liu, Ning Zhou, Yankun Zhang, Huimin Liu, Chong Xu, Yueqing Wu, Jing Zhang

**Affiliations:** Department of Respiratory and Critical Care Medicine, Tianjin Medical University General Hospital, Tianjin 300000, China; wangjuantj@163.com (J.W.); gghbtjll@163.com (L.L.); zn3355@163.com (N.Z.); lzykuun@163.com (Y.Z.); lymssdj@163.com (H.L.); xcxcxcily@163.com (C.X.); wuyueqing1006@163.com (Y.W.)

**Keywords:** SARS-CoV-2, long COVID, chronic cough, pulmonary function, chest imaging, epidemiology, risk factors

## Abstract

**Objective**: To investigate the epidemiology, clinical characteristics, and potential risk factors of chronic cough following SARS-CoV-2 infection. **Methods**: A total of 1434 patients with post-COVID-19 cough were categorized into acute, subacute, and chronic subgroups by cough duration, with clinical data analyzed across subgroups. Questionnaire surveys were conducted in chronic cough patients, followed by an 18–21-month follow-up. **Results**: 1. Significant intergroup differences were observed among the three groups in: the number of patients with rhinitis and/or pharyngitis history, cough with chest tightness, cough with pharyngeal symptoms, and sensitivity to irritating odors and cold air. 2. The chronic group had a significantly lower platelet count but higher eosinophil and basophil percentages than the acute group. 3. The chronic group showed significantly lower values than the subacute group in multiple pulmonary function indices: FVC, FEV1, FEV1/FVC, PEF, MEF25, MEF75, MEF50, MMEF75/25, MEF75%, MEF50%, MEF25%, MMEF75/25%, DLCO, and DLCO%. 4. Chest CT findings: the chronic group had significantly lower rates of infected lesions, cord-like opacities, and ground-glass shadows than the acute group, but a higher rate of micro-nodules than the subacute group. 5. At follow-up, the cough and non-cough groups differed significantly in nighttime cough scores and the proportion of cough with chest tightness, as well as in pulmonary function parameters: FVC, FEV1, PEF, PEF%, MEF75, DLCO, RV% and TLC. 6. Binary logistic regression analysis identified the nocturnal cough symptom score and cough accompanied by chest tightness as independent factors influencing persistent cough 18–21 months after SARS-CoV-2 infection. **Conclusions**: Patients with pre-existing upper airway inflammation, laryngeal symptoms, chemical hypersensitivity, elevated eosinophil/basophil percentages, and pulmonary micro-nodules are more likely to develop chronic post-COVID cough, presenting with partial ventilatory impairment and diffusing capacity impairments.

## 1. Introduction

Since December 2022, following the adjustment of China’s prevention and control policies for the coronavirus disease 2019 (COVID-19) outbreak, the country has witnessed a surge in infections dominated by the Omicron variant. Characterized by a short incubation period, mild clinical manifestations, and rapid transmission, Omicron has resulted in relatively few severe cases. However, the potential long-term symptoms induced by Omicron infection have raised growing concerns [1]. As defined by the World Health Organization (WHO), “long COVID” refers to the onset or persistence of symptoms within three months after SARS-CoV-2 infection, with such symptoms lasting for at least two months and remaining unexplained by alternative diagnoses [2].

Cough is one of the most common and prominent symptoms of SARS-CoV-2 infection. In some patients, it may progress to subacute or even chronic cough, significantly impairing their quality of life. To date, research on cough associated with COVID-19, particularly that caused by the Omicron variant, remains limited, leaving numerous critical questions to be addressed [3].

Extensive studies on long COVID, both domestically and internationally, have indicated a high prevalence of cough in long COVID syndrome. Nevertheless, there is a notable scarcity of research focusing on post-COVID-19 cough, especially chronic cough. This study therefore conducts a retrospective investigation of outpatients with chronic cough following SARS-CoV-2 infection. A targeted questionnaire was developed to explore the clinical characteristics of such chronic cough and analyze its association with relevant laboratory indicators during onset and remission. Ultimately, this research aims to provide a theoretical reference and empirical basis for clinical diagnosis and treatment.

## 2. Materials and Methods

### 2.1. Overview of Research Methods

This study adopted a retrospective research approach, enrolling patients with post-SARS-CoV-2 infection cough who attended the Respiratory Outpatient Clinic. Patients were stratified into subgroups based on the duration of their cough, and clinical data of each subgroup at the time of consultation were analyzed. Additionally, a questionnaire survey was administered to patients with chronic cough following SARS-CoV-2 infection, with follow-up assessments conducted in subsequent stages.

Questionnaire design: The questionnaire was developed through a systematic review of the existing literature on cough, consisting of two sections. The first section collected general demographic information on participants, including gender, age, smoking history, height, weight, allergic history, and past medical history. The second section focused on infection-related symptoms, such as the onset time of cough, accompanying symptoms, cough severity, potential triggering or exacerbating factors, and medications that relieved cough. Cough severity was evaluated using the Visual Analog Scale (VAS) and cough symptom scores. The survey was conducted in the Respiratory Outpatient Clinic, where patients completed the questionnaire either by scanning a QR code via mobile devices or filling out paper-based forms. The questionnaires were completed sequentially by outpatients after investigators had collected their medical history. All participants were uniformly informed of the purpose and significance of the survey to ensure informed consent.

### 2.2. Ethics Approval and Consent to Participate

This study was reviewed and approved by the Medical Ethics Committee of the General Hospital of Tianjin Medical University, with the approval number IRB 2023-YX-219-01. All procedures performed in this study were in accordance with the ethical standards of the Declaration of Helsinki.

Informed consent and permission for the publication of medical images were taken from the patient.

### 2.3. Study Population

From 1 January to 30 June 2023, a total of 1498 visits by patients with post-COVID-19 cough were recorded at the Respiratory Outpatient Clinic of Tianjin Medical University General Hospital. For patients with multiple visits, the duration of cough was determined based on the chief complaint documented during their last visit. Ultimately, 1434 patients with post-COVID-19 cough were included in the study.

Inclusion criteria: (1) SARS-CoV-2 infection; (2) presenting to the clinic with cough as the primary complaint, which developed subsequent to SARS-CoV-2 infection. Exclusion criteria: (1) Patients with poor general status or complex, severe comorbidities that would prevent their participation in follow-up assessments; (2) individuals under 14 years of age, pregnant women, and lactating women.

Questionnaire collection: A total of 524 questionnaires were collected from outpatients with post-COVID-19 cough. After excluding those with a cough duration of less than 8 weeks, 453 questionnaires from patients with post-COVID-19 chronic cough were ultimately included.

Inclusion criteria. Specific inclusion criteria for chronic cough cohort: (1) SARS-CoV-2 infection; (2) presence of chronic cough as a prominent post-infection symptom, with a duration exceeding 8 weeks after SARS-CoV-2 infection. Specific exclusion criteria for chronic cough cohort: (1) Cough duration of less than 8 weeks following SARS-CoV-2 infection; (2) a history of pre-existing respiratory diseases potentially causing chronic cough, including but not limited to chronic obstructive pulmonary disease, asthma, rhinitis, and pharyngitis; (3) meeting the aforementioned general exclusion criteria (poor general status, severe comorbidities precluding follow-up, or being under 14 years of age, pregnant, or lactating).

Diagnostic criteria for SARS-CoV-2 infection: (1) Positive nucleic acid test or antigen test report for SARS-CoV-2 during the acute infection period; (2) presence of acute-phase symptoms during the peak of infection (December 2022), such as fever, cough, sore throat, myalgia, fatigue, etc., with some patients possibly experiencing nasal congestion, runny nose, loss of smell or taste [3]. Since not all patients underwent testing during the acute phase, meeting either of the above criteria is sufficient for inclusion.

The diagnostic criteria for cough associated with SARS-CoV-2 infection are specified as follows. Acute cough due to SARS-CoV-2 infection: presents during the acute phase of infection, with a duration of 2–3 weeks. Subacute cough due to SARS-CoV-2 infection: persists beyond the resolution of the acute infection phase, with a duration ranging from 3 to 8 weeks. Chronic cough due to SARS-CoV-2 infection: continues for more than 8 weeks after the resolution of acute-phase symptoms, which reflects the persistent nature of cough in certain cases of SARS-CoV-2 infection.

### 2.4. Assessment of Covariates

General information includes gender, age, smoking history, past medical history, and other relevant details. Cough-related indicators are as follows: duration of cough after SARS-CoV-2 infection; presence of accompanying symptoms with cough; cough symptom scores; and medication efficacy. Routine blood indicators: white blood cell count (WBC), red blood cell count (RBC), hemoglobin (Hb), platelet count (PLT), neutrophil percentage (Neu%), lymphocyte percentage (Lymph%), monocyte percentage (Mon%), eosinophil percentage (Eos%), basophil percentage (Bas%), neutrophil count (Neu#), lymphocyte count (Lymph#), monocyte count (Mon#), eosinophil count (Eos#), and basophil count (Bas#). Pulmonary function indices: forced expiratory volume in 1 s (FEV1), forced vital capacity (FVC), FEV1/FVC ratio, percentage of predicted FEV1 (FEV1%), maximum expiratory flow at 50% of forced vital capacity (MEF50%), maximum expiratory flow at 25% of forced vital capacity (MEF25%), ratio of maximum expiratory flow at 75% to 25% of forced vital capacity (MEF75/25%), diffusing capacity of the lung for carbon monoxide (DLCO SB), ratio of residual volume to total lung capacity (RV% TLC), fractional exhaled nitric oxide (FeNO), and results of the bronchodilator test. Chest CT indices: presence of infectious lesions, ground-glass opacities, irregular lines, subpleural lines, interlobular septal thickening, reticular patterns, solid lesions, and traction bronchiectasis.

### 2.5. Statistical Analysis

Data analyses were performed using SPSS 27.0 software. For continuous variables, normally distributed data were expressed as mean ± standard deviation. Comparisons among three groups were conducted using one-way ANOVA, with post hoc pairwise comparisons applied for further analysis; non-normally distributed data were presented as quartiles (P25, P50, P75). Comparisons among three groups were performed using the Kruskal–Wallis H test.

For categorical variables, data were described as counts (percentages). Comparisons among three groups were carried out using the R × C chi-square test or Fisher’s exact test, with an initial statistical significance threshold of *p* < 0.05. Logistic regression analysis was employed to identify independent risk factors for post-SARS-CoV-2 chronic cough. Receiver operating characteristic (ROC) curves were constructed to evaluate the diagnostic value of relevant indicators and determine their optimal cut-off points. GraphPad Prism 10.1.2 software was used for graphical visualization.

## 3. Results

### 3.1. Clinical Data

Demographic and clinical characteristics of the 1434 enrolled participants are summarized in Table 1. The mean age of the participants was 49.03 years, with 66.9% being female. Statistically significant differences were observed among the acute, subacute, and chronic groups in terms of the number of patients with a history of rhinitis and/or pharyngitis, as well as those with cough accompanied by chest tightness, cough accompanied by pharyngeal symptoms, and sensitivity to irritating odors and cold air. Furthermore, pairwise comparisons were conducted, and the results are illustrated in (Table 1).

### 3.2. Routine Blood Test Results

A subset of participants underwent routine blood tests at the time of consultation. For patients with post-SARS-CoV-2 infection cough, the platelet counts in the acute, subacute, and chronic groups were 279 (246–326) × 10^9^/L, 244 (209–301) × 10^9^/L, and 252 (221–301) × 10^9^/L, respectively, with a statistically significant difference observed among the three groups (*p* = 0.006). The eosinophil percentages in the acute, subacute, and chronic groups were 1.10% (0.60–1.60%), 1.60% (0.90–2.30%), and 1.70% (1.00–2.80%), respectively, showing a significant difference across the three groups (*p* = 0.001). The basophil percentages in the acute, subacute, and chronic groups were 0.40% (0.30–0.50%), 0.50% (0.30–0.60%), and 0.50% (0.30–0.70%), respectively, with a statistical difference noted among the three groups (*p* = 0.005). No statistical significance was found in the remaining routine blood parameters, including white blood cells, red blood cells, lymphocytes, neutrophils, and monocytes.

In subsequent pairwise comparisons, platelet counts differed significantly between the acute and subacute groups (*p* = 0.002) and between the acute and chronic groups (*p* = 0.002). Eosinophil percentages showed a statistically significant discrepancy between the acute and subacute groups (*p* = 0.002) and between the acute and chronic groups (*p* = 0.002). Basophil percentages exhibited a significant difference between the acute and subacute groups (*p* = 0.01) and between the acute and chronic groups (*p* = 0.001).

### 3.3. Pulmonary Function Test Results

**Figure 1:**
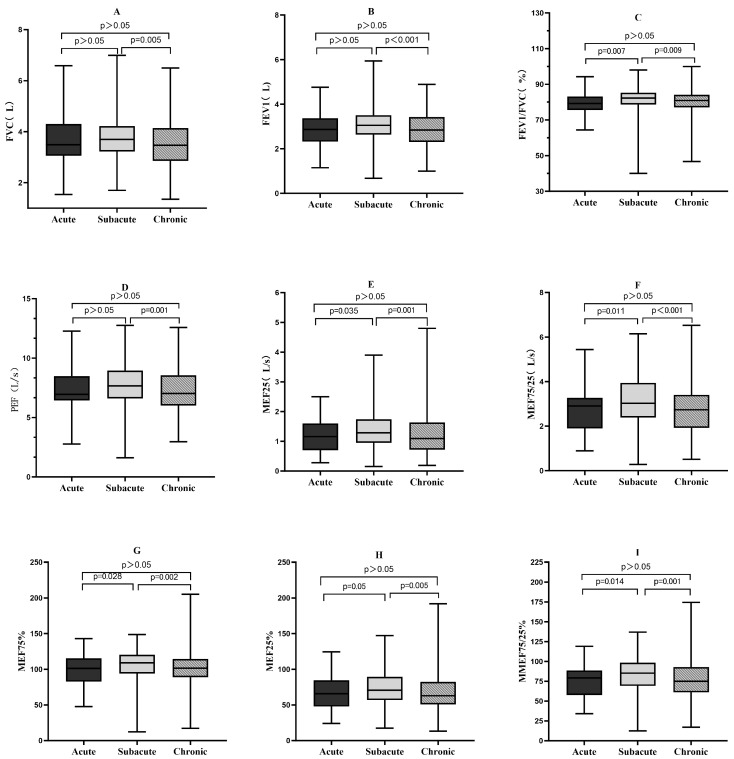

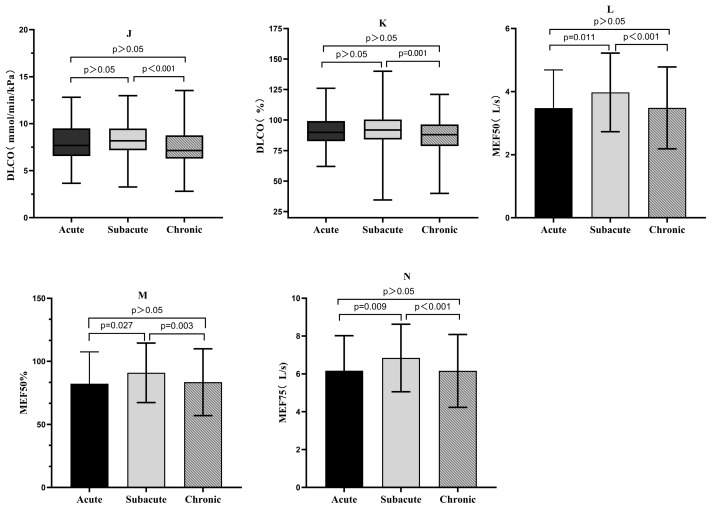
Comparison of differences in lung function indices between subgroups. (**A**) FVC; (**B**) FEV1; (**C**) FEV1/FVC %; (**D**) PEF; (**E**) MEF25; (**F**) MMEF75/25; (**G**) MEF75%; (**H**) MEF25%; (**I**) MMEF75/25%; (**J**) DLCO; (**K**) DLCO%, (**L**) MEF50; (**M**) MEF50%; (**N**) MEF75.

Of the 1434 participants, 469 underwent pulmonary function tests. Patients with post-SARS-CoV-2 cough showed statistically significant differences in FVC, FEV1, FEV1/FVC ratio, PEF, MEF25, MEF75, MEF50, MMEF75/25, MEF25%, MEF50%, DLCO and DLCO%. Further pairwise comparisons revealed statistically significant differences in all these indicators between the subacute and chronic groups (Figure 1).

### 3.4. Chest CT Findings

Of the 1434 participants, 854 underwent chest CT imaging. Of these, 712 patients showed abnormalities in chest CT reports, such as bronchitis, increased interstitial markings, linear opacities, nodular shadows, ground-glass opacities, etc. Among them, 72 patients had pre-existing abnormalities detected in tests conducted prior to COVID-19 infection. Statistically significant differences were observed among the three groups in patients with post-SARS-CoV-2 infection cough who had chest CT findings of infectious lesions, cord-like shadows, micro-nodules, and ground-glass opacities at the time of consultation. Subsequent pairwise analyses were performed using the partitioned chi-square test, with the significance level adjusted to *p* = 0.0167. For infectious lesions on chest CT in post-SARS-CoV-2 infection cough patients, statistically significant differences were observed between the acute and subacute groups (*p* < 0.001) and between the acute and chronic groups (*p* < 0.001). Regarding cords and stripe shadows on chest CT, a statistically significant difference was identified between the acute and chronic groups (*p* = 0.003), and between the subacute and chronic groups (*p* = 0.003). For tiny nodules on chest CT in post-SARS-CoV-2 infection cough patients, a statistically significant difference was noted between the subacute and chronic groups (*p* = 0.002). In terms of ground-glass opacities on chest CT, statistically significant differences were observed between the acute and subacute groups (*p* < 0.001), between the acute and chronic groups (*p* = 0.002), and between the subacute and chronic groups (*p* = 0.006).

### 3.5. Baseline Characteristics of Chronic Cough Following SARS-CoV-2 Infection

A questionnaire survey was conducted among 453 patients with chronic cough after SARS-CoV-2 diagnosis; the mean age was 44.74 years (median, 41 years), with 64.0% being female. Cough symptoms assessed by the Visual Analog Scale (VAS) had a quartile of 60 (40–78) points, daytime cough scores averaged 3 (2–3) and nighttime scores averaged 1 (1–2). Sixty-three per cent of patients had cough with phlegm, 71.5% had cough with chest tightness and wheezing, 48% reported no significant effect of medications on cough, while 23.6% found cough suppressants and expectorants effective; 84.2% denied a smoking history, 63.4% had received the COVID-19 vaccine, and 50.8% had no prior health issues or medical history.

### 3.6. Pulmonary Function in Patients with Chronic Cough Following SARS-CoV-2 Infection

A total of 453 patients with chronic cough following SARS-CoV-2 infection participated in an outpatient questionnaire survey, with their pulmonary function test data collected and analyzed; 233 pulmonary function reports were collected, showing that 64.8% of these patients had normal ventilatory function and 35.2% had abnormal ventilatory function (17.6% with small airway dysfunction, 12.0% with obstructive ventilatory dysfunction, 4.3% with restrictive ventilatory dysfunction). Of 209 pulmonary diffusion function results, 63.6% were normal and 36.4% showed reduced diffusion function; 88.5% of patients had normal residual volume–total volume ratios and 11.5% had elevated ratios; 103 patients underwent bronchodilator tests, with a positive rate of 10.68%.

### 3.7. Risk Factors for Persistent Cough 18–21 Months After SARS-CoV-2 Infection

A follow-up was conducted 18–21 months after questionnaire collection, with a median follow-up time of 19 (18–19) months post-questionnaire completion. A total of 233 patients completed the follow-up, including 41 still reporting cough and 192 without cough at follow-up. Loss to the follow-up was due to an inability to contact patients or patient refusal. Analysis of baseline data at follow-up showed that the symptom score was significantly higher in patients with persistent cough [2 (1–3.5)] than in those without cough [1 (0–2)] (*p* = 0.026). Additionally, a significant difference was observed in the number of patients with cough at follow-up (28 cases) versus those without (162 cases) (*p* = 0.016). Among patients with cough accompanied by chest tightness at initial consultation, 28% still reported cough at follow-up, while 75% did not, with a statistically significant difference (*p* = 0.022).

**Figure 2:**
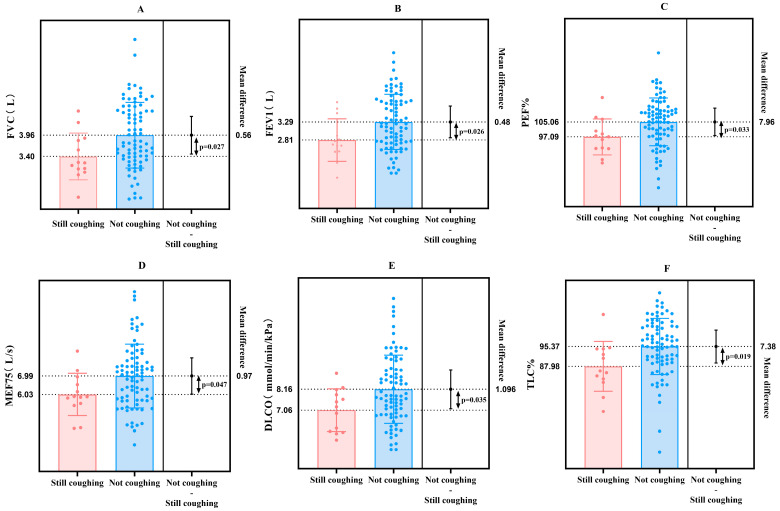

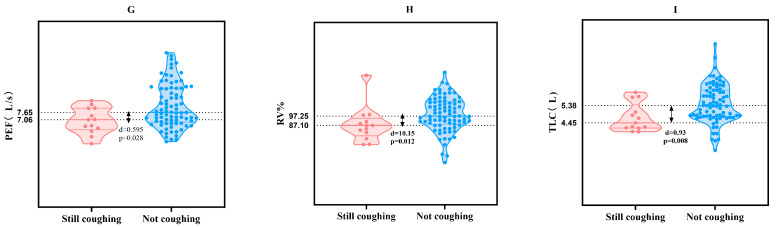
Lung function analysis between subgroups at follow-up. (**A**) FVC; (**B**) FEV1; (**C**) PEF%; (**D**) MEF75; (**E**) DLCO; (**F**) TLC%; (**G**) PEF; (**H**) RV%; (**I**) TLC.

Analysis of pulmonary function data collected during follow-up visits revealed statistically significant differences in FVC, FEV1, PEF, PEF%, MEF75, DLCO, RV%, TLC, and TLC% between patients with persistent cough and those without cough at follow-up after SARS-CoV-2 infection (Figure 2).

**Figure 3:**
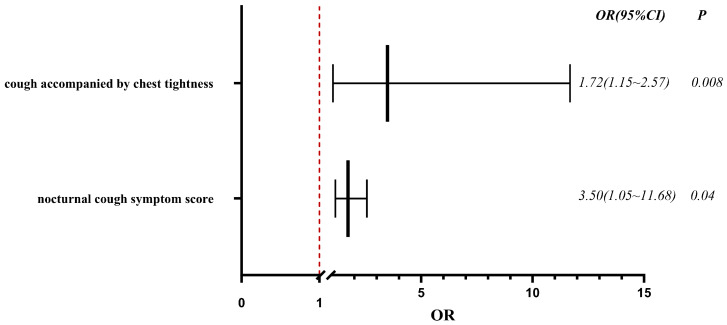
Forest plot of logistic regression analysis.

Binary logistic regression analysis, with adjustment for confounding factors, identified high nocturnal cough symptom scores and cough accompanied by chest tightness as independent predictors of persistent cough at 19 months after SARS-CoV-2 infection (Figure 3). A higher nocturnal cough symptom score was associated with an increased likelihood of unresolved chronic cough, particularly when accompanied by chest tightness. Specifically, each one-point increase in the nocturnal cough symptom score was linked to a 72% higher prevalence of cough at 19 months post-infection. Patients with cough accompanied by chest tightness were 3.5 times more likely to have persistent cough at 19 months than those without chest tightness.

### 3.8. ROC Curve Analysis Was Performed to Evaluate Persistent Cough at 18–21 Months After SARS-CoV-2 Infection

**Figure 4:**
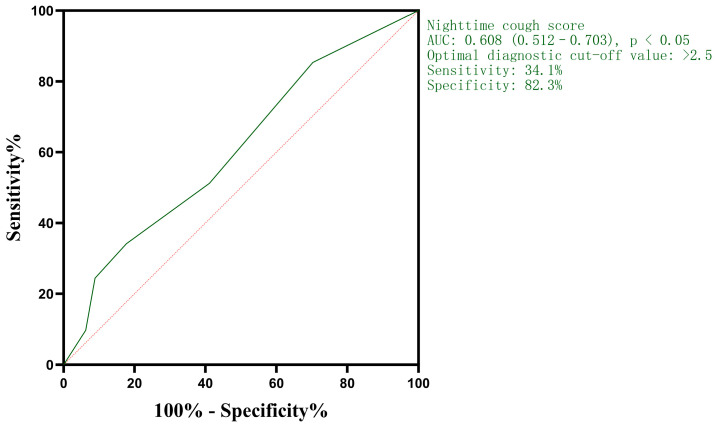
Diagnostic value of nocturnal cough symptom score in patients still coughing after 18 to 21 months of SARS-CoV-2 infection. The red line is the reference line.

An ROC curve was constructed to assess the diagnostic value of nocturnal cough symptom scores for persistent cough at 18–21 months post-infection, with the x-axis representing (1-specificity) and the y-axis representing sensitivity. For patients with post-SARS-CoV-2 chronic cough, the nocturnal cough symptom score showed diagnostic value for cough persistence at 18–21 months, with an area under the curve (AUC) of 0.608. ROC curve analysis determined an optimal diagnostic cut-off value of 2.5, corresponding to a diagnostic accuracy of 60.8% and a maximum Youden’s index of 0.164 (Figure 4).

## 4. Discussion

Cough may persist for weeks or months following SARS-CoV-2 infection. Studies have shown that 20–30% of patients may develop chronic cough, with 2.5% still experiencing cough symptoms one year after infection [4]. Similarly to other post-infectious cough syndromes, cough in long-term SARS-CoV-2 infection cases may be associated with hypersensitivity of sensory nerves, particularly the vagus nerve [5]. Research data indicates that many individuals recover from the long-term effects of SARS-CoV-2 infection within 1–2 months, while a small proportion takes longer to achieve full recovery [6]. A questionnaire survey, which grouped individuals based on the presence of post-infection cough, found that gender and smoking status had statistically significant effects on cough after SARS-CoV-2 infection [7]. A multicenter study conducted in three public hospitals in Spain showed that the prevalence of cough one year after hospitalization for SARS-CoV-2 infection was 2.5%. Among factors such as gender, age, and comorbidities, no clear risk factors associated with post-infection chronic cough were identified [8]. A national cohort study of patients with post-SARS-CoV-2 infection whose cough persisted for over 8 weeks revealed that chronic cough was more prevalent in females, with the most common symptoms being sputum production and dyspnea [9]. A cross-sectional survey in Ethiopia demonstrated a significant correlation between age and the development of pulmonary complications (e.g., chronic cough, dyspnea, increased respiratory rate, and oxygen desaturation) three months after SARS-CoV-2 infection. Participants younger than 40 years were found to have a 77.3% lower likelihood of developing post-infection pulmonary complications compared to those older than 60 years [10]. A Korean study found that patients with post-SARS-CoV-2 infection cough were younger and had a shorter cough duration compared to those with chronic cough following non-SARS-CoV-2 infections [11].

This study focused on patients with cough following SARS-CoV-2 infection. The results showed no significant differences in gender or smoking history between subgroups of post-SARS-CoV-2 infection cough patients, which may be related to variations between subgroups and the establishment of control groups. The questionnaire survey revealed that patients with post-SARS-CoV-2 chronic cough were predominantly female, with a mean age of 44.74 years. Among these patients, 63.6% had productive cough, and 71.5% experienced chest tightness and wheezing. These results are consistent with existing studies. In this study, the severity of post-SARS-CoV-2 chronic cough was assessed using symptom scores. The interquartile range of Visual Analog Scale (VAS) scores was 60 (40–78), with daytime cough symptom scores of 3 (2–3) and nighttime cough symptom scores of 1 (1–2).

Eosinophils are widely recognized as a key marker of allergic inflammation, and eosinopenia has been proposed as an indicator of severe early SARS-CoV-2 infection. This may be attributed to viral inhibition of eosinophil production or induction of eosinophil apoptosis [12]. A retrospective study in Israel found that total leukocyte counts continued to decline 7–8 months following SARS-CoV-2 infection. During the acute phase, this decrease was primarily driven by reduced lymphocytes, eosinophils, and basophils, alongside increased monocytes. In the months after the acute phase, the decline in leukocyte counts was mainly attributed to decreased absolute numbers of neutrophils, monocytes, and basophils [13]. The results of the present study revealed no significant differences in most indices among the three subgroups, except for platelet count, eosinophil percentage, and basophil percentage. Compared with the subacute and chronic subgroups, the acute cough subgroup had the highest platelet count and the lowest eosinophil and basophil percentages after SARS-CoV-2 infection, which may be associated with the inflammatory response during the acute infection phase. The observed pattern of hematological changes in this study not only reflects the characteristics of systemic inflammatory response during the acute phase of SARS-CoV-2 infection, but also suggests that the inflammatory mechanisms of chronic-phase cough may differ from those of classical eosinophilic airway diseases. These findings provide new peripheral blood evidence for the immune-inflammatory features of post-COVID-19 cough.

Changes in lung function are often characterized by restrictive patterns, which correlate with the severity of the initial disease course and improve over time. Notably, decreases in diffusion function can persist for up to one year in longer observation periods [5]. A national cohort study of patients with SARS-CoV-2 pneumonia, conducted three years after hospital discharge, indicated a significant reduction in residual air volume. However, no significant differences in spirometric parameters, total lung capacity, or diffusion parameters were observed between patients with long-term SARS-CoV-2-related sequelae and controls [14]. A domestic cohort study revealed that chronic cough following SARS-CoV-2 infection was accompanied by lung function abnormalities, including obstructive or restrictive pulmonary ventilation dysfunction in some patients and small airway abnormalities in approximately half of the patients. Additionally, 71 cases (82.6%) had a positive bronchial provocation test, and 49 cases (47.1%) received a new asthma diagnosis. A foreign study found that among 151 outpatients with cough and/or dyspnea lasting at least eight weeks after SARS-CoV-2 infection, 40 patients (26.5%) had a positive bronchodilator test [15]. In a prospective multicenter study in the Netherlands, 86% of adults hospitalized with new-onset respiratory infection and abnormal imaging findings underwent home spirometry six months after discharge. The mean baseline forced vital capacity (FVC) was 3.25 L; six months after discharge, FVC had increased by 19.1%, with lung function showing a linear improvement over time [16]. Lower FEV1 and FVC values are associated with more frequent occurrence of symptoms such as dyspnea, wheezing episodes, nocturnal awakening, and sputum production or mucus expectoration. In chronic obstructive pulmonary disease (COPD), lower FEV1 values are more closely linked to more severe symptoms, whereas this is not necessarily the case in asthma [17].

The results of this study showed that, compared with the subacute cough group, patients in the chronic cough group after SARS-CoV-2 infection had significantly lower FVC, FEV1, FEV1/FVC, PEF, MEF25, MEF75, MEF50, MMEF75/25, MEF75%, MEF50%, MEF25%, MMEF75/25%, DLCO, and DLCO%. These results are consistent with those of previous studies. The present study found that patients with chronic cough after SARS-CoV-2 infection were more likely to exhibit decreased diffusion function. Additionally, it revealed a reduction in ventilation function in these patients, suggesting that the chronic disease course may have a broader impact on lung function in post-SARS-CoV-2 infection patients. Patients with chronic cough after SARS-CoV-2 infection showed lung function abnormalities, including impaired ventilation and diffusion function. This study suggests that the pattern of pulmonary function impairment in patients with chronic cough following SARS-CoV-2 infection exhibits certain characteristic features. It differs from common airway-originated cough (e.g., CVA) and is distinct from simple upper airway or reflux-related cough, while partially overlapping with physiological changes involving parenchymal or interstitial lung involvement.

The literature reports indicate that 13–27% of patients develop fibrotic lesions, such as reticular opacities and traction bronchiectasis, 3–4 months after SARS-CoV-2 infection. These lesions are typically the most prominent and irreversible changes [5]. A cohort study conducted in Wuhan with a median follow-up period of 38.5 months showed that some patients developed diffusion abnormalities in lung function 6 months after SARS-CoV-2 infection. Abnormal chest CT findings were most commonly ground-glass opacities (GGO), followed by irregular lines [18]. Another cohort study revealed that some patients with SARS-CoV-2 pneumonia had residual abnormal lung CT findings 3 years after discharge. The most common were irregular cords, followed by bronchiectasis and subpleural lines, though these were insufficient to diagnose interstitial lung abnormalities (ILAs) [16]. A study of hospitalized patients with SARS-CoV-2 infection in Wuhan showed that cough (53.3%) was a common clinical manifestation. Thoracic CT scans performed approximately seven days after admission revealed abnormal findings in 375 patients (93.3%), with ground-glass opacities in 72.6% of cases, solid lung lesions in 30.9%, and pleural effusion in 5.2% [19]. A retrospective observational study conducted in France revealed that one-quarter of patients with chronic cough exhibited abnormalities on chest CT scans: bronchiectasis, nodules, and bronchial wall thickening were the most common findings [20]. In the present study, abnormal lung imaging findings in patients with chronic cough after SARS-CoV-2 infection included micro- and small nodules (45.7%), bronchiectasis (24.5%), ground-glass opacities (22.4%), cord-like shadows (12.2%), infectious lesions (9.4%), and interstitial lesions (4.1%). The chronic cough group showed lower rates of infectious lesions, cord-like shadows, and ground-glass opacities on chest CT, but a higher rate of micro- and small lung nodules. Consistent with previous studies, the results of this study indicate that abnormal lung imaging findings persist after SARS-CoV-2 infection. However, micro- and small nodular shadows were the most prevalent in both the acute and chronic groups, which may affect the interpretation of lung CT scans. The imaging patterns of patients with chronic cough following SARS-CoV-2 infection in this study differ not only from those of the general healthy population but also from the expected imaging manifestations of most common etiologies of chronic cough. This finding, on one hand, supports the possibility of unique subclinical pulmonary imaging changes in post-COVID cough, and on the other hand, suggests that imaging evaluation holds certain diagnostic significance in this population. Additionally, we observed that a small subset of patients exhibited abnormal findings on pulmonary CT prior to SARS-CoV-2 infection. Although new abnormalities were detected on chest CT post-infection, this finding further underscores that chest imaging findings cannot be entirely attributed to SARS-CoV-2 infection.

A study conducted in Wuhan with a median follow-up time of 8.1 months showed that among patients discharged after SARS-CoV-2 infection, those with post-infectious cough experienced more severe and frequent coughing and had a higher prevalence of chronic respiratory diseases during hospitalization. Multifactorial logistic regression analysis revealed that digestive symptoms and smoking during hospitalization were significantly associated with post-SARS-CoV-2 infection cough [21]. A domestic study analyzing the airway microbiota in nasopharyngeal swabs, nasal lavage, and induced sputum samples from patients with chronic cough following SARS-CoV-2 infection found that changes in the microbiota of the upper and lower respiratory tracts may be related to cough severity and symptom progression in these patients, and that staphylococci in nasopharyngeal swab samples could serve as a predictive factor for the relief of cough symptoms [22]. A study of children with long COVID in Shanghai showed that 143 (51.6%) out of 277 children had chest CT abnormalities after SARS-CoV-2 infection, among whom 64 (23.1%) developed long COVID pneumonia symptoms. The most common chest CT abnormality was vascular texture thickening, and there was no significant correlation between chest CT abnormalities and the development of long COVID pneumonia [23]. This study had a median follow-up time of 19 (18–19) months. Patients were grouped based on whether they still reported cough symptoms at follow-up, and differences in general information and pulmonary function between the two groups were analyzed. After adjusting for confounding factors via binary logistic regression analysis, the results showed that nocturnal cough symptom scores and cough accompanied by chest tightness were independent factors influencing cough persistence at 18–21 months after SARS-CoV-2 infection. ROC curve analysis indicated that the nocturnal cough symptom score had diagnostic value for persistent cough at 18–21 months in patients with post-SARS-CoV-2 chronic cough. Notably, pulmonary function indices were not identified as independent influencing factors in this study, and this result requires confirmation through further research. However, the nighttime cough score was not a strong predictor, suggesting that persistent cough is multifactorial.

However, we must acknowledge that in the follow-up phase, only 233 out of 453 patients with chronic cough (approximately 51%) completed the long-term follow-up. If there is a systematic discrepancy between the number of patients who completed the follow-up and those who were lost to the follow-up, this may introduce bias into the results.

All patients in this study were from a single hospital and were primarily middle-aged and Chinese, so the results may differ in other populations (e.g., Western populations, children, etc.).

This study also has several limitations. First, clinical data of patients with cough following SARS-CoV-2 infection were collected by reviewing outpatient medical records, which may have led to information errors or omissions. Furthermore, since the data is collected ex post from medical records, there is a risk of information bias—certain variables (symptom details, medical history) may be recorded inconsistently. Second, the analysis was limited to intragroup comparisons based on cough duration after SARS-CoV-2 infection, with no case–control group established to explore similarities and differences among patients with post-SARS-CoV-2 infection cough, those with cough from non-SARS-CoV-2 causes, and patients without cough after SARS-CoV-2 infection. Third, this study relied on telephone follow-ups, which precluded comprehensive assessments and re-evaluations of laboratory tests. Finally, for questionnaire participants, only pulmonary function data were collected, with no routine blood test or imaging data obtained.

## 5. Conclusions

In summary, this study concludes that patients with a history of rhinitis and/or pharyngitis, cough accompanied by pharyngeal symptoms, sensitivity to irritating odors, and elevated eosinophil and basophil percentages are more likely to develop chronic cough following SARS-CoV-2 infection. These patients also exhibit partial impairment pulmonary ventilation and diffusion function, with a high incidence of micro- and small pulmonary nodules detected on lung CT scans. Patients with post-SARS-CoV-2 chronic cough who have higher nocturnal cough scores and cough accompanied by chest tightness are more likely to experience persistent cough at 18–21 months, and nocturnal cough symptom scores were found to have diagnostic value for such persistence.

## Figures and Tables

**Table 1:** Clinical data of patients with acute cough, subacute cough and chronic cough after SARS-CoV-2 infection.

	Acute Group (n = 248)	Subacute Group (n = 705)	Chronic Group (n = 481)	*p*-Value
Age (years)	48.92 ± 16.07	48.63 ± 15.27	49.68 ± 15.65	0.52
Female, %	165 (66.5%)	454 (64.4%)	340 (70.7%)	0.07
Smoking (current smokers), %	70 (28.2%)	230 (32.6%)	138 (28.7%)	0.241
Rhinitis and/or pharyngitis	18/248 (7.3%) ^a^	148/705 (21%) ^b^	95/481 (19.8%) ^b^	0.001
History of chronic cough and/or cough-prone conditions	6/248 (2.4%)	16/705 (2.3%)	5/481 (1.0%)	0.25
Underlying lung disease	31/248 (12.5%)	62/705 (8.8%)	36/481 (7.5%)	0.078
Cardiovascular and metabolic diseases	29/248 (11.7%)	73/705 (10.4%)	54/481 (11.2%)	0.822
Other diseases	31/248 (12.5%)	105/705 (14.9%)	66/481 (13.7%)	0.622
Accompanying symptoms, %				
Cough and sputum	143/248 (57.7%)	386/705 (54.8%)	254/481 (52.8%)	0.457
Chest tightness	30/248 (12.1%) ^a,b^	95/705 (13.5%) ^b^	34/481 (7.1%) ^a^	0.002
Shortness of breath	29/248 (11.7%)	102/705 (14.5%)	85/481 (17.7%)	0.084
Pharyngeal symptoms	31/248 (12.5%) ^a^	130/705 (18.4%) ^a^	126/481 (26.2%) ^b^	<0.001
Irritating odors, cold air sensitivity	13/248 (5.2%) ^a^	89/705 (12.6%) ^b^	94/481 (19.5%) ^c^	<0.001
Treatment effect, %				
Medication not effective	15/248 (6.0%)	53/705 (7.5%)	29/481 (6.0%)	0.563
Relievable	27/248 (10.9%%)	81/705 (11.5%)	40/481 (8.3%)	0.200

^a,b,c^ The same letter indicates no statistically significant difference between the two groups, while different letters indicate a statistically significant difference between groups (*p* < 0.0167). ‘Cardiovascular and metabolic diseases’ refer to those with one or more of these three diseases: hypertension, diabetes mellitus, and coronary heart disease.

## Data Availability

The original contributions presented in this study are included in the article. Further inquiries can be directed to the corresponding author.
